# NOXA-Induced Alterations in the Bax/Smac Axis Enhance Sensitivity of Ovarian Cancer Cells to Cisplatin

**DOI:** 10.1371/journal.pone.0036722

**Published:** 2012-05-09

**Authors:** Chao Lin, Xin-yu Zhao, Lei Li, Huan-yi Liu, Kang Cao, Yang Wan, Xin-yu Liu, Chun-lai Nie, Lei Liu, Ai-ping Tong, Hong-xin Deng, Jiong Li, Zhu Yuan, Yu-quan Wei

**Affiliations:** 1 State Key Laboratory of Biotherapy and Cancer Center, West China Hospital, West China Medical School, Sichuan University, High Technological Development Zone, Chengdu, China; 2 Cancer Center, Chengdu Military General Hospital, Chengdu, China; 3 Department of Pathogen Biology, Chengdu Medical College, Chengdu, China; Henry Ford Health System, United States of America

## Abstract

Ovarian cancer is the most common cause of death from gynecologic malignancy. Deregulation of p53 and/or p73-associated apoptotic pathways contribute to the platinum-based resistance in ovarian cancer. NOXA, a pro-apoptotic BH3-only protein, is identified as a transcription target of p53 and/or p73. In this study, we found that genetic variants of Bcl-2 proteins exist among cisplatin-sensitive and -resistant ovarian cancer cells, and the responses of NOXA and Bax to cisplatin are regulated mainly by p53. We further evaluated the effect of NOXA on cisplatin. NOXA induced apoptosis and sensitized A2780s and SKOV3 cells to cisplatin *in vitro* and *in vivo*. The effects were mediated by elevated Bax expression, enhanced caspase activation, release of Cyt C and Smac into the cytosol. Furthermore, gene silencing of Bax or Smac significantly attenuated NOXA and/or cisplatin-induced apoptosis in chemosensitive A2780s cells, whereas overexpression of Bax or addition of Smac-N7 peptide significantly increased NOXA and/or cisplatin-induced apoptosis in chemoresistant SKOV3 cells. To our knowledge, these data suggest a new mechanism by which NOXA chemosensitized ovarian cancer cells to cisplatin by inducing alterations in the Bax/Smac axis. Taken together, our findings show that NOXA is potentially useful as a chemosensitizer in ovarian cancer therapy.

## Introduction

Ovarian cancer is the most common cause of death from gynecologic malignancy [1]. Although there are some improvements, the long-term survival remains poor due to dose-dependent toxicity, eventual tumor recurrence and emergence of drug-resistant disease. To overcome these hurdles, investigations have increasingly focused on new therapeutic strategies: modulation of cellular chemosensitivity, reversing tumor resistance, and increasing therapeutic effects of chemotherapy [2]. Emerging evidences suggest that deregulated apoptosis pathway is a major contributor to tumor initiation, progression, and development of acquired resistance to anticancer therapies [3], [4].

As a common genetic event in ovarian carcinoma, p53 mutation is associated with resistance to platinum-based chemotherapy [5]. Recent reports have shown that p73, a member of p53 family proteins, is a key regulator of apoptosis susceptibility to cisplatin (Cis) in A2780 ovarian cancer cells [6], [7], and that p73-dependent transcriptional program is an important contributor to the chemosensitivity pathway in BRCA1-deficient ovarian carcinoma cells [8], indicating some mechanisms affecting p73 expressions and functions may contribute to the development of resistance to cisplatin-induced apoptosis in ovarian cancer cells [7]. All these observations suggest that deregulation of p53-dependent and/or p73-associated apoptotic pathways may contribute to the platinum-based resistance in ovarian cancer. Thus, restoration of the p53 and/or p73 pathway by activating themselves or their downstream targets may be an attractive avenue to improve efficacy of anticancer therapies.

NOXA was first identified as a transcriptional target of p53 [9], and recently it was also shown to be regulated transcriptionally by p73 [8]. Like many Bcl-2 family proteins that translocate to mitochondria and modulate mitochondrial function, NOXA translocates to mitochondria and then leads to cytochrome C (Cyt C) release and caspase-9 activation, and, ultimately, leading to cell death [10], [11]. NOXA functions through Bax and/or Bak to induce apoptosis in some cancer cells such as Hela human epithelial cervical cancer cells [9], melanoma cells [11], MCF-7 human breast cancer cells [12], etc. Furthermore, a recent report showed a therapeutic potential of NOXA in treating human breast cancer [12]. However, the role of NOXA in the therapeutic responses of ovarian cancer cells to platinum-based anticancer drugs remains unclear.

In this work, we first selected cisplatin-sensitive (A2780s, IGROV1 and OAW42) and -resistant (A2780cp, OVCAR-3 and SKOV3) human ovarian cancer cell lines to test the expression variations of prosurvival and proapoptotic Bcl-2 family proteins. Then, we examined cisplatin-induced expression levels of p53, p73, p21^waf1/cip1^, NOXA and Bax in several human ovarian cancer cell lines with different p53 status including A2780s (p53 wild-type, p53 WT), SKOV3 (p53 double deletion mutant, p53-/-), OVCAR-3 (harboring mutant p53 R248Q) and A2780cp (containing p53 wild-type gene sequence but showing loss of p53 function) [13], [14]. We found that p53, p73, p21^waf1/cip1^, NOXA and Bax were significantly induced by cisplatin in p53-wild type A2780s cell line, but in other three p53-mutant ovarian cancer cell lines, the expressions of p73, p21^waf1/cip1^, NOXA and Bax remained unchanged. Furthermore, the responses of NOXA and Bax to cisplatin are regulated mainly by p53 other than p73 in ovarian cancer cell lines. Considering the major regulatory function of p53 on NOXA and Bax, two p53 and/or p73 downstream target genes, we further selected the p53 double deletion mutant SKOV3 cell line as a model of intrinsic resistance [15]–[17], and the p53-wild type A2780s cell line, which was derived from a untreated patient with primary ovarian carcinoma [17], [18], as a model of intrinsic chemosensitivity, to evaluate the effect of NOXA on the chemotherapeutic efficacy of cisplatin in A2780s and SKOV3 ovarian cancer models *in vitro* and *in vivo*. We found that NOXA induces apoptosis independently of p53 in both A2780s and SKOV3 cells, and that elevated expression of NOXA can enhance sensitivity of ovarian cancer cells to cisplatin through the alterations in the Bax/Smac axis. To our knowledge, we provide new evidence for the potential application of NOXA as a chemosensitizer in ovarian cancer therapy.

## Materials and Methods

### Ethics Statement

All studies involving mice were approved by the Institutional Animal Care and Treatment Committee of State Key Laboratory of Biotherapy, Sichuan University.

### Materials

The Smac-N7 peptide (AVPIAQKPRQIKIWFQNRRMKWKK) were purchased from Calbiochem, Inc. (San Diego, CA). It was modified to be cell permeable by linkage of the COOH-terminal lysine to the arginine of an Antennapedia homeodomain 16-mer peptide via a proline linker. The primary antibodies for western blotting are as follows: Anti-Bcl-2 (sc-130307), anti-Bcl-x_L_ (sc-8392), anti-Mcl-1 (sc-69839), anti-NOXA (sc-30209), anti-p53 (DO-1), Anti-p73 (H-79), anti-p21^Waf1/Cip1^ and anti–β-actin (Santa Cruz, CA); anti-Bax N-20 (sc-493); anti–caspase-3, anti–cleaved caspase-3, anti–caspase-9 and its cleaved form (Cell Signaling Technology, Danvers, MA); anti-Smac (clone FKE02, R&D Systems, Minneapolis, MN); anti-Cyt C (sc-13156), cytochrome oxidase subunit IV (Molecular Probes).

### Plasmids

Both pcDNA3.1(pc3.1) and pcDNA3.1 plasmid encoding Human NOXA gene (pcDNA3.1-hNOXA, pc3.1-hNoxa) were purified by two rounds of passage over EndoFree columns (Qiagen, Chatsworth, CA), as reported previously [19]. pc3.1-Bax constructs were generated according to our previous methods [20].

### Gene silencing with small interfering RNAs

Small interfering RNA (siRNA) oligonucleotides were purchased from Dharmacon (Lafayette, CO) with sequences targeting Bax (5′-AACUGAUCAGA ACCAUCAUGG-3′) and Smac (5′-AACCCUGUGUGCGGUUCCUAU-3′). For Bax and Smac shRNA construction, the Bax and Smac siRNA were cloned into the pSilencer 2.1-U6 hygro plasmid.

### Cell culture and transfection

A2780s, IGROV1, OAW42, A2780cp, OVCAR-3 and SKOV3 human ovarian cancer cell lines were purchased from ATCC (Manassas, VA) and grown in DMEM or RPMI 1640 culture containing 10% fetal bovine serum (FBS), respectively, at 37°C in a humidified atmosphere containing 5% CO_2_. Transfection was performed with Lipofectamine™ 2000 according to the manufacturer's instruction.

### Treatments of cells in the *in vitro* experiments

A2780s and SKOV3 cells were treated as follows: Control, the cells were left untreated and harvested when cultured for 72 h. pc3.1 (empty vector), cells were transfected with pc3.1, and then harvested at 48 h posttransfection. hNOXA, cells transfected with pc3.1-hNOXA were harvested at 48 h posttransfection. Cisplatin, cisplatin (5 µg/ml) was added when cells were cultured for 48 hours. 24 hours later, cells were harvested. hNOXA plus cisplatin, cells transfected with pc3.1-hNOXA were added cisplatin (5 µg/ml) at 24 h posttransfection. Twenty-four hours later, cells were harvested.

For gene silencing or overexpression of Bax and Smac, the corresponding siRNAs or plasmids were co-transfected with pc3.1/pc3.1-NOXA plasmids into A2780s or SKOV3 cells. The cells treated above were added cisplatin for additional 12 hours at 12 h post-transfection, and then harvested at 24 h post-transfection.

### Detection of hNOXA expression *in vitro* and *in vivo*

For detection of hNOXA expression *in vitro*, A2780s cells were treated according to the schedules described above. The harvested cells were used to detect hNOXA expression by RT-PCR and western blot, respectively. The tumor tissues were collected for detecting hNOXA expression *in vivo* by RT-PCR. The primers used for amplification of hNOXA and GAPDH were as follows: NOXA-F 5′-AAGAACGCTCAACCGAGC-3′and NOXA-R 5′-GGTTCCTGAGCAGAAGAGT-3′; GAPDH-F 5′-AATCCCATCACCATCTTCC-3′ and GAPDH-R 5′-CAT CACGCCACAGTTTCC-3′.

### Cell viability and apoptosis assays

Cell viability was assessed by MTT assay [21]. Apoptosis was assessed as follows: detection of DNA fragmentation with the Cell Death Detection ELISA kit (Roche Diagnostics), western blot analysis of caspase activation, measurement of apoptotic cells by flow cytometry (PI staining for sub-G1) and Hoechst 33258 staining. The Cell Death Detection ELISA quantified the apoptotic cells by detecting the histone-associated DNA fragments (mono- and oligo-nucleosomes) generated by the apoptotic cells [20], [22].

### Flow cytometric analysis and hoechst 33258 staining

Flow cytometric analysis was performed to identify sub-G1 cells/apoptotic cells and to measure the percentage of sub-G1 cells in hypotonic buffer as described previously [23]. Hoechst 33258 staining was performed accordingto the manufacturer's instructions.

### Semiquantitative RT-PCR

Total RNA was isolated using Trizol reagent (Invitrogen). Semiquantitative RT-PCR was done to amplify NOXA and GAPDH.

### Subcellular fractionation and western blot

Subcellular fractionation was done to obtain mitochondrial and cytosolic fractions as previously described [24]. Total cell lysates were prepared as described previously [25]. Total cell lysates and subfractionation lysates were used for western blot analysis.

### Animal tumor models and treatment

A2780s and SKOV3 cells (2×10^6^ cells) were implanted s.c. into the right flanks of 6- to 8-week-old female nude mice, respectively. To explore the therapeutic efficacy of NOXA plus cisplatin, we treated the mice on day 10 after the implantation of tumor cells, when tumor diameter reached ∼5 mm in diameter. The mice were randomly divided into 5 groups (5 mice per group) and treated with: (a).100 µl PBS; (b).10 µg pc3.1 plasmid/30 µg liposome complexes in 100 µl PBS; (c).10 µg pc3.1 -hNoxa plasmid/30 µg liposome complexes in 100 µl PBS; (d).100 µl of 0.1 mg cisplatin (5 mg/kg body weight); (e).10 µg pc3.1-hNoxa plasmid/30 µg liposome complexes in 100 µl PBS and 100 µl of 0.1 mg cisplatin. The mice were treated with DNA-liposome complex by intravenous administration *via* the tail vein twice a week, and cisplatin by intraperitoneal route once a week for 4 weeks. Tumor volumes were calculated by the following formula: tumor volume (mm^3^) = 0.52×length (mm)×width (mm)×width (mm) [26]. The tumor tissues were collected for TUNEL experiments.

### Terminal deoxynucleotidyl-transferase-mediated dUTP nick end labeling (TUNEL) analysis

TUNEL was performed with an In situ Cell Death Detection Kit (Roche). Cell apoptosis was quantified by determining the percentage of positively stained cells for all of the nuclei in 20 randomly chosen fields/section at 200× magnification. Slides of the apoptosis studies were quantified in a blind manner by two independent reviewers two different times.

### Statistical analyses

The statistical analysis was performed with SPSS software (version 17.0 for Windows). All the values were expressed as means ± SD. ANOVA and Tukey–Kramer multiple comparison test were used in comparisons. Survival curves were constructed according to the Kaplan-Meier method. Statistical significance was determined by the log-rank test. *p* value<0.05 were considered significant. Error bars represent the SEM unless otherwise indicated.

## Results

### Genetic variants among the cisplatin-sensitive and -resistant ovarian cancer cells

**Figure 1:**
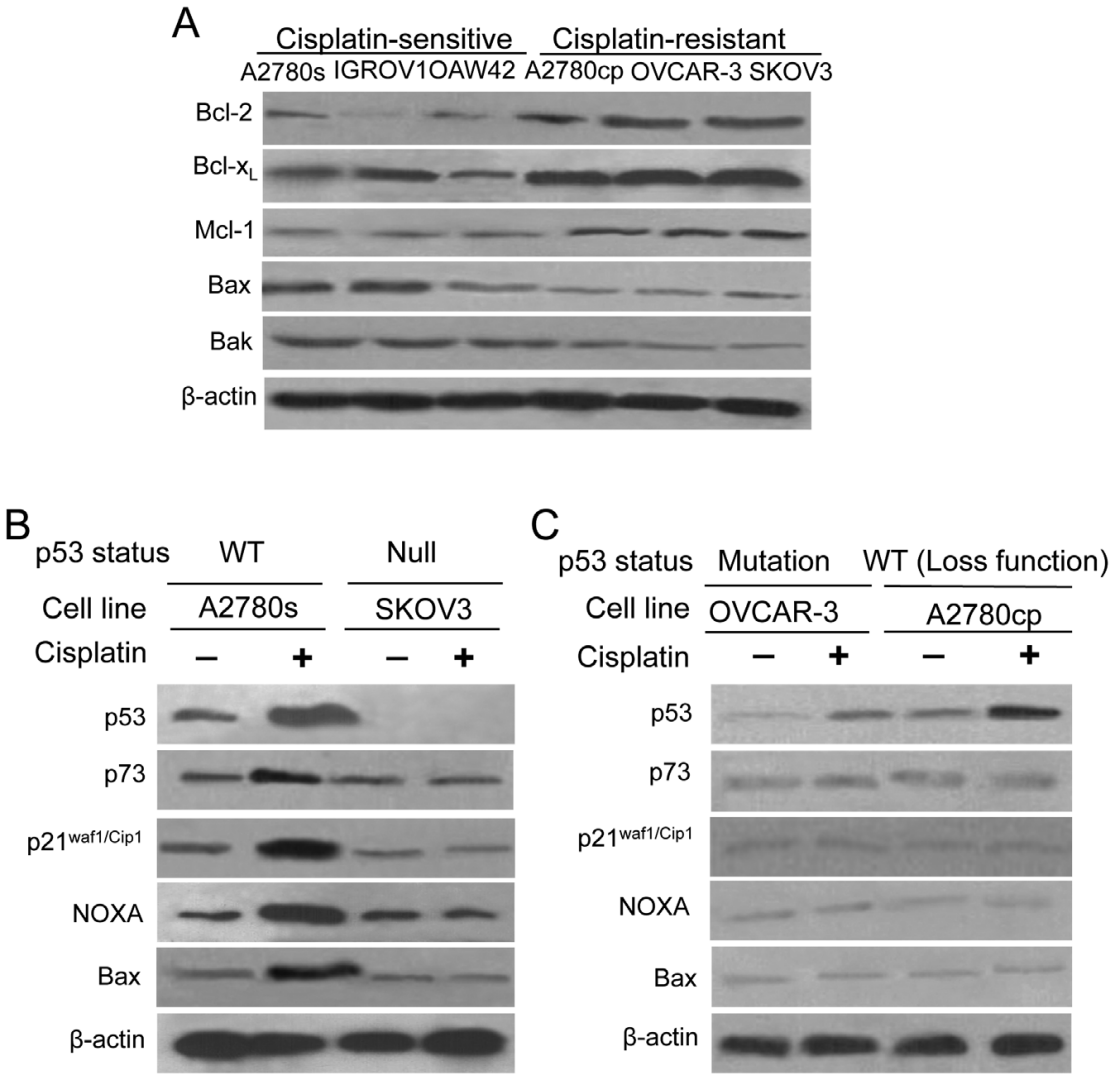
Genetic variants among the cisplatin-sensitive and -resistant ovarian cancer cells. (**A**) Western blotting was performed for the expression variations of prosurvival Bcl-2, Bcl-x_L_ and Mcl-1 and proapoptotic Bak and Bax proteins among the cisplatin-sensitive (A2780s, IGROV1 and OAW42) and -resistant (A2780cp, OVCAR-3 and SKOV3) ovarian cancer cells. β-actin was used as a loading control. (**B**) A2780s (p53 WT) and SKOV3 (p53 -/-) cells were treated with cisplatin (5 µg/mL) for 24 hours and analyzed for the expression of p53, p73, p21^waf1/cip1^, NOXA and Bax by Western blotting. β-actin was used as a loading control. (**C**) OVCAR3 (harboring mutant p53 R248Q) and A2780cp (containing p53 wild-type gene sequence but showing loss of p53 function) cells were treated with cisplatin (5 µg/mL) for 24 hours and analyzed for the expression of p53, p73, p21^waf1/cip1^, NOXA and Bax by Western blotting. β-actin was used as a loading control.

Western blotting analysis showed that cisplatin-sensitive (A2780s, IGROV1 and OAW42) cell lines express relatively low endogenous levels of Bcl-2, Bcl-x_L_ and Mcl-1 while cisplatin-resistant (A2780cp, OVCAR-3 and SKOV3) cell lines were on the contrary. In contrast to prosurvival Bcl-2 family proteins, the levels of proapoptotic Bak and Bax in A2780s, IGROV1 and OAW42 cell lines are higher than those in A2780cp, OVCAR-3 and SKOV3 cell lines (Figure 1A).

We further examined cisplatin-induced expression levels of p53, p73, p21^waf1/cip1^, NOXA and Bax in several human ovarian cancer cell lines with different p53 status including A2780s (p53 WT), SKOV3 (p53-/-), OVCAR-3 (harboring mutant p53 R248Q) and A2780cp (containing p53 wild-type gene sequence but showing loss of p53 function). All the indicated cells were treated with 5 µg/ml cisplatin for 24 hr. As shown in figure 1B and C, p53, p73, p21^waf1/cip1^, NOXA and Bax were found to be significantly induced by cisplatin in p53-wild type A2780s cell line, but in other three p53-mutant cisplatin-resistant OVCAR-3, A2780cp and SKOV3 cell lines, the expressions of p73, p21^waf1/cip1^, NOXA and Bax remained unchanged. Furthermore, the level of endogenous Bax in cisplatin-resistant OVCAR-3, A2780cp and SKOV3 cell lines is very low (figure 1B and C). These results indicate that the responses of NOXA and Bax to cisplatin are regulated mainly by p53 other than p73 in ovarian cancer cell lines.

### Reduced viability of ovarian cancer cells *in vitro* by hNOXA and cisplatin

**Figure 2:**
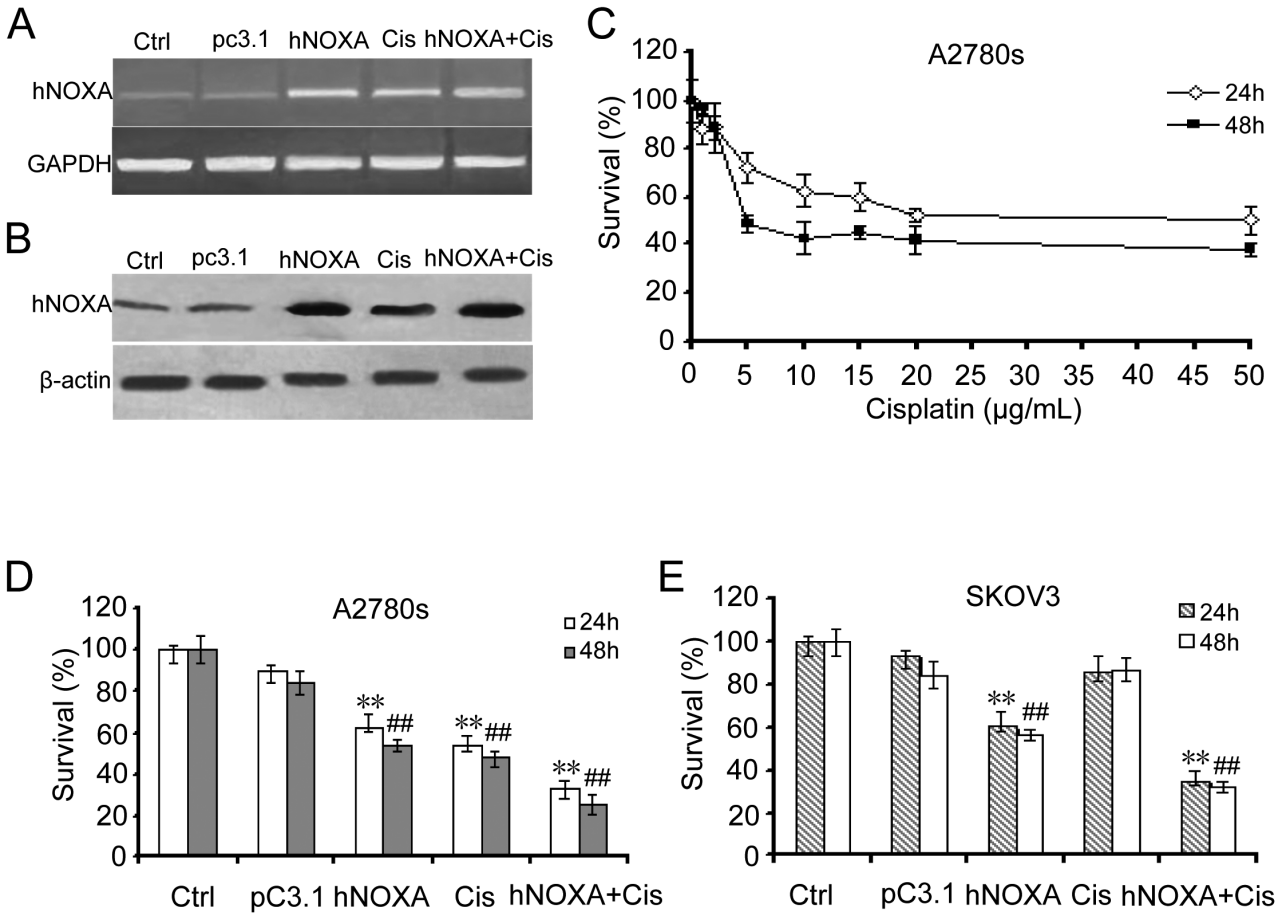
Reduced viability of ovarian cancer cells In Vitro by hNOXA and cisplatin. (**A**) RT-PCR analysis of hNOXA expression *in vitro* after transfection of A2780s cells. GAPDH was used as a loading control. (**B**) Western blotting analysis of hNOXA expression *in vitro* after transfection of A2780s cells. β-actin was used as a loading control. (**C**) The treatment of cisplatin at indicated concentrations and periods reduced A2780s cell viability, showing that the dose of IC_50_ ranged from 5 µg/ml to 10 µg/ml. (**D**) The treatment of hNOXA plus cisplatin reduced A2780s cell viability more significantly than the treatment of hNOXA alone or cisplatin alone did. Significant differences compared with the control group (24 h, **P<0.001; 48 h, ^##^P<0.001). (**E**) The treatment of cisplatin alone had little effect on survival of SKOV3 cells, and the combination of hNOXA plus cisplatin reduced SKOV3 cell viability more significantly than the treatment of hNOXA alone or cisplatin alone did. Significant differences compared with the control group (24 h, **P<0.001; 48 h, ^##^P<0.001). Percentage of survival was calculated. Results are shown as means ± SD of three wells and triplicate experiments. In each experiment, the medium-only treatment (untreated) indicates 100% cell viability.

The pro-apoptotic function of NOXA and lack of NOXA induction in intrinsically cisplatin-resistant SKOV3 (p53-/-) ovarian cells prompted us to investigate whether overexpression of NOXA suppresses ovarian cancer cell growth. Overexpression of hNOXA in transfected A2780s cells was confirmed by RT-PCR (Figure 2A) and western blotting analysis (Figure 2B), respectively. Considering that NOXA functions downstream of the p53-mediated apoptotic pathway, and that the cytotoxic action of cisplatin is mediated by DNA damage, which, in turn, transactivates target genes (e.g.p53AIP, PUMA, NOXA) to cause apoptosis, we predicts that elevated NOXA expression can sensitize ovarian cancer cells to cisplatin. To test this hypothesis, we first treated A2780s cells with cisplatin at indicated concentrations, with a 24- or 48-hr interval, and found that the dose of IC_50_ of cisplatin ranged from 5 µg/ml to 10 µg/ml (Figure 2C). Then, we treated cells with cisplatin at a suboptimal dose (5 µg/ml), with a 24/48-hour interval, according to the various schedules as described in Meterials and Methods. After treatment, viability of cells was determined by MTT assay. As shown in figure 2D, compared with the control, either hNOXA or cisplatin significantly reduced A2780s cell viability by 41%/47% (P<0.001) and 43%/49% (P<0.001), respectively. hNOXA plus cisplatin very significantly reduced A2780s cell viability by 68%/76% (P<0.001). In p53-deficient SKOV3 cells, compared with the pc3.1 control, hNOXA also significantly reduced cell viability (P<0.001) while cisplatin showed only a slight, but not statistically significant effect on SKOV3 cell growth (24 h, p = 0.874; 48 h, p = 0.921). However, the combination of hNOXA and cisplatin very significantly reduced SKOV3 cell viability by 65%/68% (P<0.001) (Figure 2E).

### Induction of apoptosis of ovarian cancer cells *in vitro* by hNOXA and cisplatin

**Figure 3:**
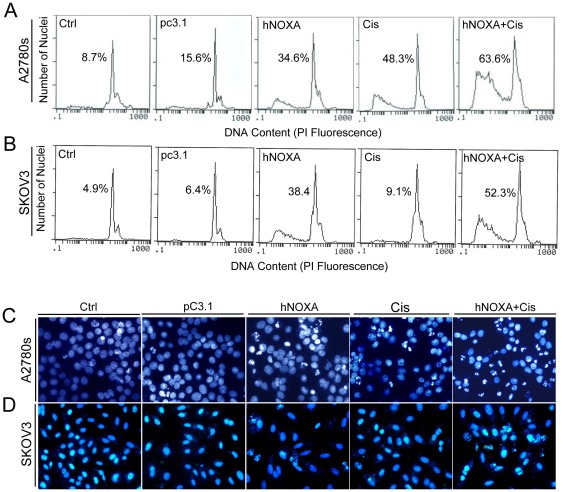
Induction of apoptosis of tumor cells *in vitro* by hNOXA and cisplatin. (**A**) Representative DNA fluorescence histograms of PI-stained cells. A2780s cells were treated with hNOXA for 24 h, then with 5 µg/ml cisplatin for an additional 24 h. A2780s cells were treated with pcDNA3.1 or hNOXA, cisplatin alone or hNOXA plus cisplatin and groups Ctrl, pc3.1, hNOXA, Cis and hNOXA+Cis correspond to these five treatments (the same as shown in the subsequent panels), with 8.7% (Ctrl), 15.6% (pc3.1), 34.6% (hNOXA), 48.3% (Cis) and 63.6% (hNOXA+Cis) sub-G1 cells (apoptotic cells), respectively, as assessed by flow cytometry. (**B**) SKOV3 cells were treated with hNOXA for 24 h, then with 5 µg/ml cisplatin for an additional 24 h. SKOV3 cells were untreated, treated with empty vector or hNOXA, cisplatin alone or hNOXA plus cisplatin, with 4.9% (Ctrl), 6.4% (pc3.1), 38.4% (hNOXA), 9.1% (Cis) and 52.3% (hNOXA+Cis) sub-G1 cells (apoptotic cells), respectively, as assessed by flow cytometry. (**C**) Normal and apoptotic nuclear morphology of A2780s cells was analyzed by Hoechst 33258 staining. A2780s cells were treated with the same conditions as described above. (**D**) Normal and apoptotic nuclear morphology of SKOV3 cells was analyzed by Hoechst 33258 staining. SKOV3 cells were treated with the same conditions as mentioned above.

The quantitative assessment of sub-G1 cells by flow cytometry was used to estimate the number of apoptotic cells. As shown in figure 3A, in cisplatin-sensitive A2780s cells, the apoptotic cells accounted for 34.6% in hNOXA-treated group versus 15.6% in pcDNA3.1-treated group and 8.7% in control group. The apoptotic cells accounted for 63.6% in the combination group versus 48.3% in cisplatin-treated group. These results suggested that either cisplatin or hNOXA alone significantly induced apoptosis of A2780s cells, and hNOXA plus cisplatin further augmented the induction of apoptosis. Similar results were obtained in intrinsically resistant SKOV3 cells except that cisplatin alone was found to have little ability to induce apoptosis of SKOV3 cells (Figure 3B).

Apoptosis was further evaluated by Hoechst 33258 staining. Similar to the above results, in both A2780s and SKOV3 cells, the number of condensed nuclei (intact or fragmented), which are characteristic of apoptosis, in the combination group were observed than that in hNOXA- or cisplatin-treated cells. There was no significantly condensed nuclei in medium only- and pc3.1-treated groups. However, it should be noticed that cisplatin-treated SKOV3 cells showed no similar apoptotic signs (Figure 3C and D).

### The sensitizing effects of NOXA are mediated by release of Cyt C and smac into the cytosol

**Figure 4:**
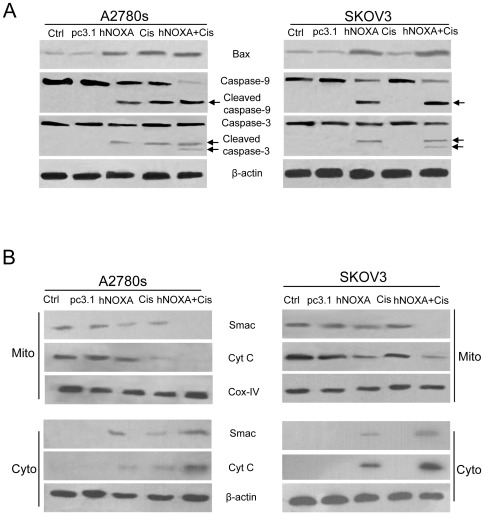
The sensitizing effects of hNOXA are mediated by enhanced caspase activation and release of Cyt C and Smac into the cytosol. (**A**) A2780s and SKOV3 cells were subjected to the indicated treatments as described in Materials and Methods. Caspase activation were analyzed by Western blotting. Arrows indicate active forms of caspases. β-actin was used as a loading control. (**B**) A2780s and SKOV3 cells were subjected to the indicated treatments as described in Materials and Methods. Release of Cyt C and Smac into the cytosol were analyzed by Western blotting. Cox-IV blots indicate mitochondrial loading controls while β-actin was used as a loading control for the cytosolic fraction.

NOXA induces apoptosis through activation of Bax and/or Bak to trigger mitochondrial dysfunction and caspase-9 activation [10], [11], [27]. As expected, in cisplatin-sensitive A2780s cells, either hNOXA or cisplatin alone resulted in significant up-regulation of Bax and activation of caspases 3 and 9, and their combination further enhanced the up-regulation of Bax and activation of the caspases (figure 4A). Furthermore, the apoptosis was accompanied by release of Cyt C and Smac into the cytosol (Figure 4B). Similar results were also obtained in intrinsically resistant SKOV3 cells, except that cisplatin alone was found not to cause the up-regulation of Bax and activation of the caspases and release of Cyt C and Smac (Figure 4A and B).

### Alterations in the Bax/Smac axis determines sensitivity of ovarian cancer cells to cisplatin

We observed that up-regulation of Bax and release of Smac into the cytosol in NOXA-treated A2780s and SKOV3 cells (Figure 4), and that cisplatin also led to Bax up-regulation and Smac release in A2780s cells (Figure 4), but in SKOV3 cells, it did not caused up-regulation of Bax (Figure 1 and 4) and release of Smac into the cytosol (Figure 4). These observations prompted us to speculate that Bax/Smac axis may be one of key determinants of chemosensitivity in cisplatin-resistant ovarian cancer cells, and that NOXA-induced alterations in the Bax/Smac Axis may enhance sensitivity of ovarian cancer cells to cisplatin. To test the speculation, we decided to investigate whether NOXA and/or cisplatin-induced apoptosis was attenuated in cisplatin-sensitive A2780s cells when treated with siRNA targeting Bax or Smac, and whether NOXA and/or cisplatin-induced apoptosis was enhanced in cisplatin-resistant SKOV3 cells when pretreated with Bax construct or Smac N7 peptide, respectively.

**Figure 5:**
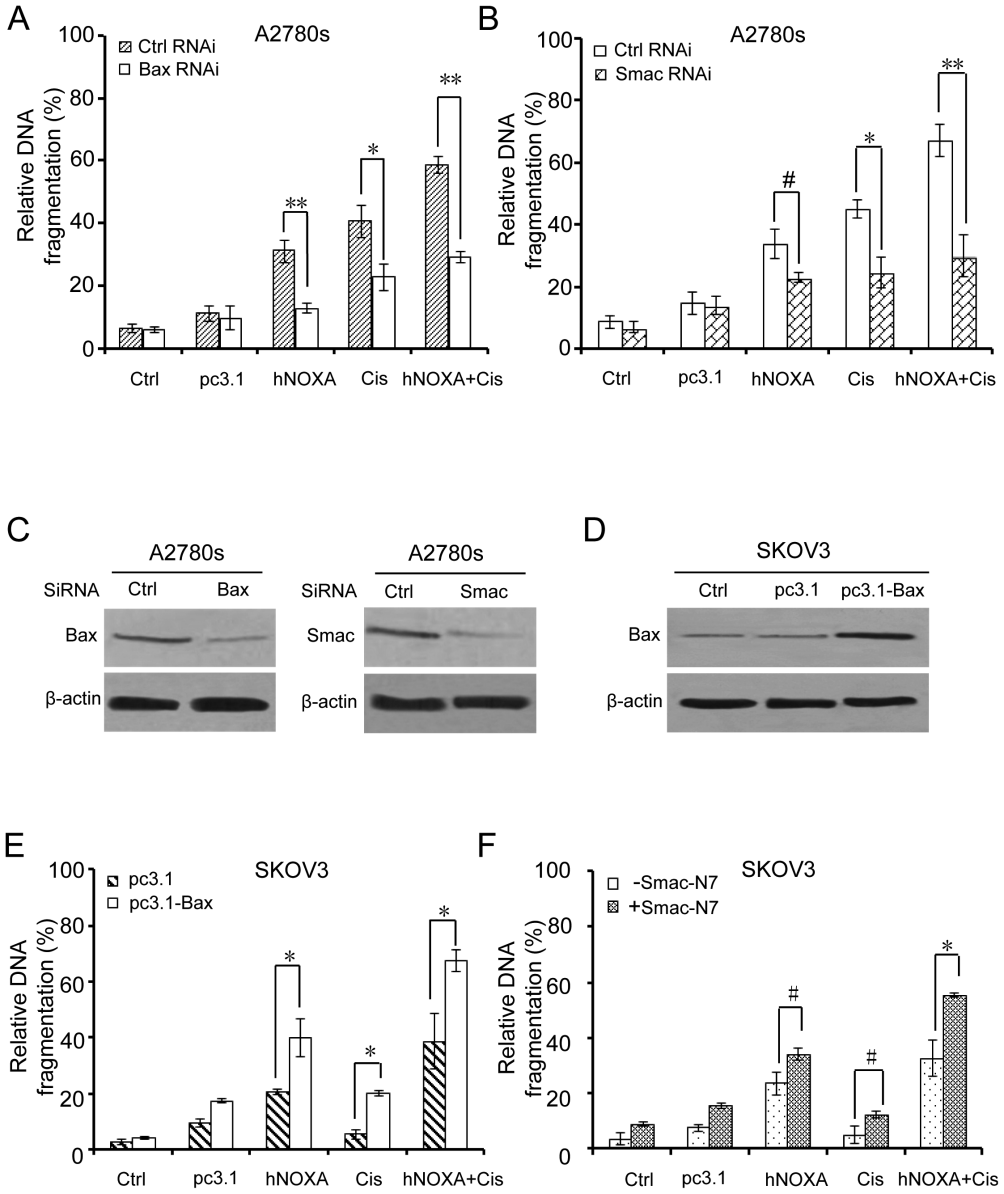
Alterations in the Bax/Smac Axis affect sensitivity of Ovarian Cancer Cells to Cisplatin. (**A**) siRNAs targeting Bax significantly attenuated NOXA and/or cisplatin-induced apoptosis in chemosensitive A2780s cells (*P<0.01; **P<0.001). (**B**) siRNAs targeting Smac significantly attenuated NOXA and/or cisplatin-induced apoptosis in chemosensitive A2780s cells (^#^P<0.05; *P<0.01; **P<0.001). (**C**) Down-regulation of Bax or Smac by Bax siRNA or Smac siRNA were confirmed by Western blot. (**D**) Overexpression of Bax was confirmed by Western blot. (**E**) SKOV3 cells were co-transfected with NOXA and pc3.1-Bax plasmids for 12 hours, followed by 5 µg/mL cisplatin treatment for additional 12 hours. Overexpression of Bax significantly increased NOXA and/or cisplatin-induced apoptosis in chemoresistant SKOV3 cells (*P<0.01). (**F**) SKOV3 cells were treated with NOXA and/or cisplatin as described above, then with 20 µmol/L Smac-N7 peptide for an additional 3 hours. Smac-N7 peptide significantly increased NOXA and/or cisplatin-induced apoptosis in chemoresistant SKOV3 cells (^#^P<0.05; *P<0.01).

As expected, Bax siRNA significantly attenuated NOXA and/or cisplatin-induced apoptosis in A2780s cells (Figure 5A). Similar results were also found in A2780s cells after treatment with siRNA targeting Smac (Figure 5B). The specificity of Bax and Smac siRNA duplexes was evaluated by analyzing Bax and Smac protein expression in A2780s cells transfected with the corresponding shRNA construction (Figure 5C). In pc3.1-Bax-transfected SKOV3 cells, overexpression of Bax, which was confirmed by western blotting analysis (Figure 5D), significantly increased NOXA and/or cisplatin-induced apoptosis (Figure 5E). Similarly, addition of an NH2-terminal Smac heptapeptide (Smac-N7) also significantly enhanced NOXA and/or cisplatin-induced apoptosis (Figure 5F).

### Enhanced antitumor efficacy of the combination of hNOXA and cisplatin in vivo

**Figure 6:**
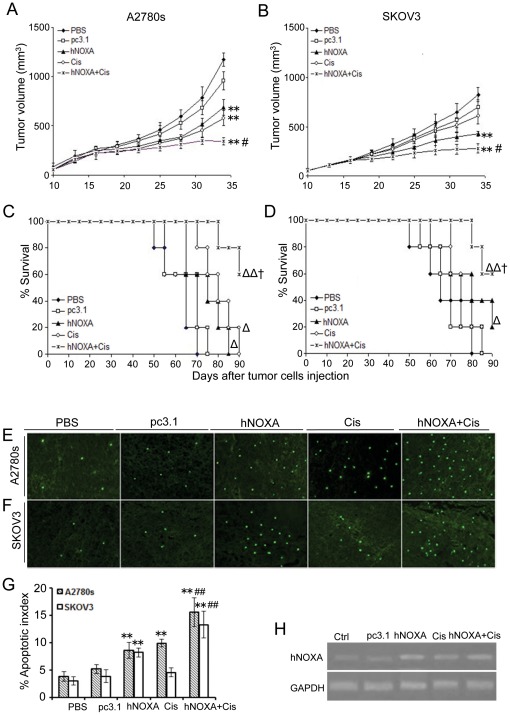
Enhanced antitumor efficacy of the combination of hNOXA and Cisplatin In Vivo (A–D) Tumor suppression and survival advantage in mice. A2780s cells (A, C) or SKOV3 cells (B, D) of 2×10^6^ were inoculated subcutaneously into female nude mice at 6–8 weeks of age. Mice (five per group) were treated with PBS, pcDNA3.1, pcDNA3.1-hNOXA, Cisplatin and pcDNA3.1-hNOXA+cisplatin. In A2780s tumor model, significant differences in tumor suppression (**P<0.001) and survival time (^Δ^P<0.05) in mice treated with hNOXA or cisplatin versus PBS and pcDNA3.1 controls; significant difference for tumors treated with hNOXA+cisplatin versus PBS and pcDNA3.1 controls (**P<0.001; ^ΔΔ^P<0.01), and significant difference for the combination therapy versus hNOXA or cisplatin monotherapy (^#^P<0.05; ^†^P<0.05). Similar results were also found in SKOV3 model, except that no significant differences in tumor suppression (*P* = 0.222) and survival time (*P* = 0.433) between cisplatin- and pcDNA3.1-treated tumors were found. (**E–F**) TUNEL staining of tumor tissues. Representative sections were taken from A2780s (E) and SKOV3 (F) tumor tissues of mice receiving PBS, pcDNA3.1, hNOXA, cisplatin and hNOXA+cisplatin. (**G**) Apoptotic index within A2780s and SKOV3 tumor tissues were counted. In A2780s model, statistically significant difference in the apoptotic index for tumors treated with hNOXA or cisplatin versus PBS and pcDNA3.1 controls (**P<0.001); significant difference for tumors treated with hNOXA+cisplatin versus the two controls (**P<0.001); and significant difference for the combination therapy versus hNOXA or cisplatin monotherapy (^##^P<0.001). Similar results were also found in SKOV3 model, except that no significant differences in the apoptotic index between cisplatin-treated tumor and pcDNA3.1-treated tumor (P = 0.981) or PBS-treated tumor (P = 0.705) were found. The apoptotic index was calculated as a ratio of the apoptotic cell number to the total cell number in each field. (**H**) RT-PCR analysis of expression of exogenous hNOXA in vivo.

Based on the *in vitro* growth-inhibitory and pro-apoptotic effects of hNOXA and cisplatin, we further examined the antineoplastic effect of hNOXA plus cisplatin on A2780s and SKOV3 tumors *in vivo*. As shown in figure 6A and B, on day 34 after implantation, the A2780s and SKOV3 tumors of mice treated with PBS reached 1174.28±70.43 and 823.82±73.27 mm^3^ in volume, respectively. The A2780s and SKOV3 tumors treated with hNOXA were significantly (A2780s model, *P*<0.001; SKOV3 model, *P*<0.001) smaller than those treated with PBS, reaching only 686.06±81.39 and 429.38±22.9 mm^3^ in volume, respectively. The combination of hNOXA and cisplatin further suppressed tumor growth such that the A2780s and SKOV3 tumors reached 342.84±38.8 and 279.27±47.16 mm^3^ in volume, respectively, which were significantly (A2780s model, *P*<0.001; SKOV3 model, *P*<0.001) smaller than control tumors, and significantly smaller than the tumors treated with hNOXA (A2780s model, *P*<0.001; SKOV3 model, p<0.05) or cisplatin (A2780s model, *P*<0.05; SKOV3 model, *P*<0.001). Cisplatin also resulted in a significant reduction in tumor volume (577.08±77.04 mm^3^) compared with control tumors (*P*<0.001) in the A2780s model. However, in SKOV3 model, no significant difference in tumor volume (605.44±80.51 mm^3^) was observed in the cisplatin-treated group compared with the pc3.1-treated group (695.57±79.28 mm^3^) (*P* = 0.222).

Survival curve analysis (Figure 6C) showed that A2780s tumor bearing mice in the PBS or pc3.1-treated groups survived less than 65 days on average. By contrast, either hNOXA or cisplatin resulted in a significant (*P*<0.05) increase in life span compared with the two control groups, with the mean survival time being 74 and 80 days, respectively. The combination of hNOXA and cisplatin further improved survival to a greater extent than the two control groups (*P*<0.01), with the mean survival time being 87 days. Except that there was no significant difference in survival time between cisplatin-treated mice and PBS-treated mice (*P* = 0.128) or pc3.1-treated mice (*P* = 0.433), similar results were also found in the SKOV3 tumor model (Figure 6D).

TUNEL was further performed to detect the apoptosis in tumor tissues. In A2780s tumor model, hNOXA and/or cisplatin increased the apoptotic rate of tumor cells when compared with PBS or empty vector (Figure 6E and G). However, no significant differences in the apoptotic index of tumors treated with the two monotherapies were found in A2780s model (P = 0.296). Similar results were also found in SKOV3 tumor model, except that no significant differences in apoptotic index were observed between cisplatin-treated tumors and pc3.1-treated tumors (P = 0.981) or PBS-treated tumors (P = 0.705) (Figure 6F and G).

The observations that hNOXA chemosensitized A2780s and SKOV3 cells to cisplatin besides its antineoplastic effect *in vivo* raised a question whether the enhanced antitumor efficacy resulted from the delivery of hNOXA via tail vein injection. To confirm whether the treatment using liposome delivery of hNOXA via tail vein injection actually gets to the tumor cells, RT-PCR was performed. As expected, *in vivo* overexpression of exogenous hNOXA was verified by RT-PCR in A2780s tumor tissues (Figure 6H), indicating that intravenous injections of pc3.1-hNOXA plasmid led to the expression of exogenous hNOXA within the tumor tissues.

## Discussion

NOXA, a “BH3-only” member of the Bcl-2 family, was shown to be a target of p53 and/or p73-mediated transactivation [10], [11]. NOXA first translocates to mitochondria and then functions through Bax and/or Bak to induce apoptosis [10], [11]. Recent studies demonstrated that NOXA could induce apoptosis of some cancer cells such as Hela epithelial cervical cancer cells [9], melanoma cells [11], MCF-7 breast cancer cells [12], and suggested a therapeutic potential in the treatment of human breast cancer [12]. However, the role of NOXA in the therapeutic responses of ovarian cancer cells to platinum-based anticancer drugs remains unclear. The current study was designed to investigate whether NOXA could induce apoptosis of ovarian cancer cells, and whether it could potentiate antineoplastic effects of cisplatin on ovarian cancer cells.

Many cancer cells express prosurvival Bcl-2 family proteins, thereby rendering cells resistant to apoptosis [28], [29]. Previous studies have shown that Bcl-2 and Bcl-x_L_ proteins appear to be involved in chemoresistance in ovarian carcinoma [30]–[32], and that reduced Bax expression is associated with cisplatin resistance in ovarian carcinoma cell systems [33]. More recently, Bcl-x_L_ and Mcl-1 were reported to be able to cooperate to protect ovarian carcinoma cells against oncogenic stress or chemotherapy-induced apoptosis [34]. Consistent with these observations, our data demonstrated that both relative high levels of Bcl-2, Bcl-x_L_ and Mcl-1 and low levels of Bak and Bax are associated with the chemoresistance of human ovarian cancer cells (Figure 1A). We further found that p53, p21^waf1/cip1^, which is indicative of a functional p53, p73, NOXA and Bax were significantly induced by cisplatin in p53-wild type A2780s cell line, but in other three p53-mutant (SKOV3, OVCAR-3 and A2780cp) ovarian cancer cell lines, the expressions of p73, p21^waf1/cip1^, NOXA and Bax remained unchanged (Figure 1B and C), indicating that the responses of NOXA and Bax to cisplatin are regulated mainly by p53 other than p73 in ovarian cancer cell lines.

Considering the major regulatory function of p53 on NOXA and Bax, we then selected the p53 double deletion mutant SKOV3 cell line as a model of intrinsic resistance, and the p53 wild-type A2780s cell line as a model of intrinsic chemosensitivity, respectively, to evaluate the effect of NOXA on the chemotherapeutic efficacy of cisplatin *in vitro* and *in vivo*. We found that overexpression of hNOXA induced apoptosis and enhanced sensitivity of both intrinsically cisplatin-sensitive A2780s and –resistant SKOV3 cells to cisplatin, as evidenced by MTT assay (Figure 2), flow cytometry analysis (Figure 3A and B), Hoechst 33258 staining (Figure 3C and D), activation of caspases 3 and 9 (Figure 4A) and release of Cyto c and Smac into the cytosol (Figure 4B). Furthermore, the *in vitro* enhanced antiproliferative and pro-apoptotic activities of hNOXA plus cisplatin on ovarian cancer cells correlates well with the *in vivo* improved antitumor efficacy. The enhanced antitumor efficacy *in vivo* was associated with the enhanced induction of apoptosis, as verified by TUNEL analysis (Figure 6).

Previous studies have shown that cisplatin-induced apoptosis can be initiated through both intrinsic and extrinsic pathways. Cisplatin induces rapid dose-dependent release of Cyt C from mitochondria to cytosol [35]. Cyt C subsequently activates the caspase cascade, eventually causing apoptotic cell death [36]. We found that cisplatin induces apoptosis of chemosensitive A2780s cells, but not chemoresistant SKOV3 cells. Furthermore, cisplatin-induced apoptosis is associated with activation of caspase 3 and 9. These observations are in agreement with previous reports that caspase 3 and 9 are important for cisplatin-induced apoptosis, and their activation is attenuated in resistant cells [37], [38].

The key regulatory proteins of mitochondria-mediated apoptotosis are the Bcl-2 family of proteins, which can either promote cell survival, as Bcl-2 and Bcl-x_L_ do, or induce cell death, as Bax and Bak do [39]. Bax plays a key role in mediating apoptosis induced by certain anti-cancer agents [40]. Our data showed that cisplatin induces up-regulation of Bax and release of mitochondrial Cyt c and Smac into cytoplasm in chemosensitive A2780s cells, but not in chemoresistant SKOV3 cells, whereas NOXA induces up-regulation of Bax and release of mitochondrial Cyt c and Smac in both A2780s and SKOV3 cells. These results is consistent with the notion that Bax exerts at least part of its activity by triggering Cyt c release from mitochondria [39].

Smac, also called direct inhibitor of apoptosis proteins (IAP)-binding protein with low pI (Diablo), was also found to be released into the cytosol of apoptotic cells [41]. Smac interacts with and antagonizes IAPs, such as XIAP, cIAP1 and cIAP2 [41]. Recently, Smac release is shown as a determinant of chemosensitivity in ovarian cancer cells [42]. A previous report has shown that TRAIL-induced apoptosis requires Bax-dependent mitochondrial release of Smac/DIABLO [39]. Our recent publication has also shown that Smac potentiates Bax activation, and that Smac-mediated Bax activation is a major molecular event in AT101-induced apoptosis in chemoresistant ovarian cancer cells [20]. These observations by us and others suggest an important role of Bax/Smac axis in the apoptosis of cancer cells.

Taken together, we speculated that NOXA enhance sensitivity of ovarian cancer cells to cisplatin by inducing alterations in the Bax/Smac Axis. The speculation was supported by our findings as follows: siRNAs targeting Bax or Smac significantly attenuated NOXA and/or cisplatin-induced apoptosis in chemosensitive A2780s cells (Figure 5A and B), whereas overexpression of Bax or addition of an NH2-terminal Smac heptapeptide (Smac-N7) significantly increased NOXA and/or cisplatin-induced apoptosis in chemoresistant SKOV3 cells (Figure 5 E and F). Our results are similar to previous reports that cisplatin-induced apoptosis in ovarian cancer cells depends on efficient Bax expression [7], and that SMAC expression and agents that mimic the IAP interacting function of SMAC sensitize human cancer cells to apoptosis induced by several anticancer agents, such as tumor necrosis factor (TNF)-related apoptosis-inducing ligand (TRAIL) and TNF alpha [43], [44].

In conclusion, our data suggest that elevated expression of NOXA can induce apoptosis independently of p53 in both cisplatin-sensitive A2780s (p53WT) and cisplatin-resistant SKOV3 (p53 -/-) ovarian cancer cells, and that it can enhance the therapeutic responses of ovarian cancer, especially the intrinsically resistant, p53 double deletion mutant ovarian cancer cells, to cisplatin by inducing alterations in the Bax/Smac axis. To our knowledge, we provide the first evidence for the potential application of NOXA as a chemosensitizer in ovarian cancer therapy.
