# Correction: NOXA-Induced Alterations in the Bax/Smac Axis Enhance Sensitivity of Ovarian Cancer Cells to Cisplatin

**DOI:** 10.1371/annotation/a863b4b3-6d4c-447a-87e3-4d631ceb7a46

**Published:** 2012-08-10

**Authors:** Chao Lin, Xin-yu Zhao, Lei Li, Huan-yi Liu, Kang Cao, Yang Wan, Xin-yu Liu, Chun-lai Nie, Lei Liu, Ai-ping Tong, Hong-xin Deng, Jiong Li, Zhu Yuan, Yu-quan Wei

The following authors should have been designated as contributing equally to the article: Chao Lin, Xin-yu Zhao, and Lei Li. 

